# Pragmatic randomised controlled trial of group psychoeducation versus group support in the maintenance of bipolar disorder

**DOI:** 10.1186/1471-244X-11-114

**Published:** 2011-07-21

**Authors:** Richard K Morriss, Fiona Lobban, Steven Jones, Lisa Riste, Sarah Peters, Christopher Roberts, Linda Davies, Debbie Mayes

**Affiliations:** 1 Professor of Psychiatry and Community Mental Health, Institute of Mental Health, University of Nottingham & Nottinghamshire Healthcare NHS Trust; 2 Senior Lecturer in Clinical Psychology, Spectrum Centre for Mental Health Research, School of Health and Medicine, Lancaster University; 3 Professor of Clinical Psychology, Spectrum Centre for Mental Health Research, School of Health and Medicine, Lancaster University; 4 PARADES Programme Manager, Department of Psychology, University of Manchester; 5 Senior Lecturer in Psychology, University of Manchester; 6 Reader in Medical Statistics, School of Medicine, University of Manchester; 7 Professor of Health Economics, University of Manchester; 8 Service User Researcher, Spectrum Centre for Mental Health Research, Lancaster University

## Abstract

**Background:**

Non-didactically delivered curriculum based group psychoeducation has been shown to be more effective than both group support in a specialist mood disorder centre in Spain (with effects lasting up to five years), and treatment as usual in Australia. It is unclear whether the specific content and form of group psychoeducation is effective or the chance to meet and work collaboratively with other peers. The main objective of this trial is to determine whether curriculum based group psychoeducation is more clinically and cost effective than unstructured peer group support.

**Methods/design:**

Single blind two centre cluster randomised controlled trial of 21 sessions group psychoeducation versus 21 sessions group peer support in adults with bipolar 1 or 2 disorder, not in current episode but relapsed in the previous two years. Individual randomisation is to either group at each site. The groups are carefully matched for the number and type of therapists, length and frequency of the interventions and overall aim of the groups but differ in content and style of delivery. The primary outcome is time to next bipolar episode with measures of the therapeutic process, barriers and drivers to the effective delivery of the interventions and economic analysis. Follow up is for 96 weeks after randomisation.

**Discussion:**

The trial has features of both an efficacy and an effectiveness trial design. For generalisability in England it is set in routine public mental health practice with a high degree of expert patient involvement.

**Trial Registration:**

ISRCTN62761948

**Funding:**

National Institute for Health Research, England.

## Background

Recurrence rates for mania and depression in bipolar disorder are high; around 50% at one year and 70% at four years [1,2]. Group psychoeducation in addition to maintenance medication is recommended by most recent bipolar disorder practice guidelines for the maintenance management of bipolar disorder [3-6]. In Barcelona, two randomised controlled trials showed that structured curriculum based group psychoeducation for up to 21 sessions increased time to relapse in all types of bipolar episode in patients who were concordant or not concordant with mood stabiliser medication compared to non-didactic group support not following a curriculum [7,8]. The gains were maintained over the next five years with marked reductions in hospitalisation and improvements in function [9]. Subgroup analysis showed improvements with psychoeducation versus control intervention in bipolar 2 disorder [10] and in bipolar disorder with or without personality disorder [11]. In Australia, two randomised controlled trials showed that 12 sessions of non-didactic curriculum based group psychoeducation reduced the rate of bipolar episode relapse compared to treatment as usual [12,13].

The trials in Barcelona were conducted in a specialist bipolar disorder service and the nature of the group support provided in the trials was not well characterised. In the United Kingdom, lay led peer support groups led by service users are more frequently found than in other European countries [14]. Although, generally peer support groups may improve self-efficacy and may be cost effective [15] their effectiveness in bipolar disorder is less certain. However, self management strategies to stay well, (developed by service users with bipolar disorder who had no bipolar episodes for at least two years) were valued as important as medication [16]. Therefore a pragmatic randomised controlled trial is needed to compare group psychoeducation close in format to the Barcelona programme with peer group support where patients identify their own learning needs. Such a trial would tease out whether the content of the Barcelona psychoeducation programme is more or less effective than the group process of bringing together people with bipolar disorder facilitated by health professionals.

The trial also permits further work to be conducted on the mechanisms of action of group psychoeducation. The Barcelona model of psychoeducation incorporates all of the recommended components of psychological treatment recommended by NICE [3] such as early warning signs [17], medication adherence [18], and maintaining a regular daily routine [19] together with psychoeducation about the disorder, and medication. Importantly the groups use the collective experiences of the group members to bring about positive changes in attitudes, knowledge and behaviour among the participants.

## Methods/Design

### Objective

To determine the clinical and cost effectiveness of joint expert patient and health professional led group psychoeducation for bipolar disorder versus unstructured peer group support.

Main research questions:

• To demonstrate that group psychoeducation is feasible and sustainable across different non-specialist sites across the English National Health Service

• To determine the clinical and cost effectiveness of group psychoeducation compared to peer group support

• To identify barriers and potential solutions to barriers to the implementation of effective group psychoeducation

### Design

**Figure 1:**
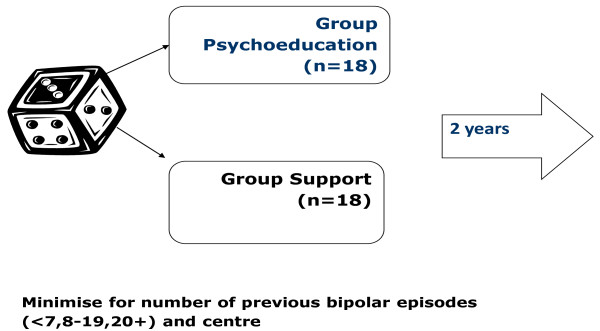
**Design of the study**.

A randomised controlled trial compares the effectiveness of:

• 21 weekly session bipolar group psychoeducation, delivered by two health professionals (nurse, psychiatrist, psychologist, occupational therapist) and an expert patient, plus treatment as usual from the psychiatrist and other health professionals versus

• 21 weekly session unstructured bipolar group support, led by peers and facilitated by two health professionals and the expert patient, plus treatment as usual for patients with bipolar disorder (Figure 1).

The trial is located in two centres (East Midlands and North West) in England, with four clinical sites in each centre. There are five waves so that the largest clinical sites run two groups each and the remaining clinical sites run one group each. The intervention is taken to different parts of a regional centre to improve access for those people wish to participate but for whom travelling distance is a practical barrier to engagement.

Consecutively eligible patients are individually randomised to either intervention with stratification by clinical site and minimisation in terms of number of previous bipolar episodes (<7, 8-19, 20+) (see Figure 1). The latter is to control for the effect of rate of bipolar episodes: relapse is up to three times greater in those with more than 20 episodes than in those with less than seven bipolar episodes [20]. In some RCTs of psychosocial interventions in bipolar disorder there may be an interaction between psychological treatment and number of previous bipolar episodes [21]. Barriers to the effectiveness of either intervention are examined qualitatively by interviewing maximum variance samples of participants and group facilitators.

### Setting

Community mental health team bases at a number of NHS Trusts located in two distinct geographical centres (East Midlands and North West) are used to ensure the generalisability of the findings. The study is also promoted at a primary care level with local family doctors being asked to display posters about the trial. Research assistants (RAs), service user researchers and community scientific officers from the Mental Health Research Network in England visit support groups such as the Manic Depression Fellowship (MDF), Poles Apart and Mood Swings to introduce the study and provide referral information.

### Target population

The target population is patients with bipolar 1 or 2 affective disorder at increased risk of further relapse (an episode in the last 24 months). In clinical practice patients at increased risk of further relapse are the targets for interventions aimed at preventing further relapse rather than patients who have been stable for several years.

### Inclusion/exclusion criteria

Inclusion criteria are:

• a SCID-DSM-IV verified diagnosis of primary bipolar disorder [22],

• at increased risk of relapse (at least one episode in the last 24 months),

• age 18 years or more.

Exclusion criteria are:

• presence of a current manic, hypomanic, mixed affective or major depressive episode currently or within the previous four weeks,

• current suicide plans or high suicide intent,

• inability or unwillingness to give written informed consent to the study.

• inability to communicate in written and verbal English to a sufficient level to allow them to complete the measures and take part in the groups.

### Baseline and Outcome Measures

At baseline interview the Structured Clinical Interview for DSM-IV (SCID) is used to assess sociodemographic features, the presence of axis 1 comorbid psychopathology [23], the presence of borderline or antisocial personality disorder [24] and the number of previous bipolar episodes. Participants are also asked at baseline how effective they think each of the two treatments is likely to be and if they have any preference as to which group they are allocated to.

The primary outcome measure is:

• time to next bipolar episode, average weekly symptom score (both established using 16-weekly SCID Longitudinal Interval Follow-up Evaluation (LIFE) interviews [25,26] to generate weekly scores of mania and depression on 1-6 scale of severity), as we have previously used [20,21].

Secondary outcome measures include:

• time to next manic-type episode (mania, hypomania or mixed affective episode) and time to next depressive episode [20,21]

• assessment of mean and variability in weekly symptoms of mania type symptoms and depression symptoms using the LIFE [20,21]

• assessment of function using the Social Adjustment Scale [27] and SOFAS [28];

• observer and self-rated measures of mood: 17 item Hamilton-GRID (HDRS) [29,30]; Bech-Raphaelson Mania Scale (MAS) [31]; Hospital Anxiety & Depression Scale (HAD) [32];

• medication adherence (Medad) [33];

• health status and related utility values using the Euroqol 5D [34,35] and health and social care costs from a broadly societal perspective.

Further measures are used to explore the process of change with treatment:

• The Hayward Stigma Questionnaire [36], an eight-item self-report questionnaire to examine if effectiveness of the groups might be related to reductions in stigma.

• The KAB (Knowledge about Bipolar Disorder). This is based on the Knowledge About Schizophrenia Inventory [37], and asks questions on knowledge about bipolar disorder found in the group psychoeducation manual. The questions were tested on groups of service users with bipolar disorder before the trial to ensure that the questions were understood and that the KAB did not have either ceiling or floor effects. The questionnaire is used to determine if differences in outcome between the two groups might be due to differences in the knowledge of bipolar disorder acquired by the participants.

• The Hypomania Interpretations Questionnaire [38], a 10-item self-report questionnaire to explore if either group changed positive self-dispositional appraisals for hypomania-related experiences.

• The Social Rhythm Metric [39,40] trait and diary forms are completed over one week. The habitual timing of 17 daily behaviours is assessed.providing information on the number, timing and frequency of occurrence of regular activities.

• The Short-Form 12 (SF-12) [41], a 12-item self-completed questionnaire evaluating eight domains of overall health (general health, role physical, physical function, bodily pain, vitality, social functioning, role emotional, and mental health) in the preceding four weeks summarised as physical component scores and mental component scores.

• The Early Warning Signs checklist [42], a 32-item and 31-item checklist of common early warning signs of depression and mania, and the timing of these signs in relation to the onset of a depressive or mania episode respectively.

• The Coping Strategies Checklist [20], a list of adaptive and maladaptive coping strategies employed with the onset of manic symptoms (40 items) [43] and depression (40 items) symptoms. The coping with depression checklist was derived from items from the Response Style Questionnaire [44], the Depression Coping Checklist [45], items from the coping with mania questionnaire [43] that were also relevant to depression according to a panel of service users from a UK service user organisation (the Bipolar Organisation: the Manic Depression Fellowship) plus four additional items suggested by these service users.

• The Brief Illness Perception Questionnaire [20,46], a 11-item self-report measure of beliefs about mood swings in bipolar disorder, each measure on a five point scale of strength of conviction.

In addition, weekly ratings of treatment fidelity for each treatment session during the 21 session treatment are taken using a short checklist designed specifically for this study. Participants are asked to provide feedback specifically on group cohesion [47] and group working alliance (WAI-S) [48] which are administered at weeks 3, 10 and 21 of follow-up.

Follow-up of each patient is for 96 weeks from randomisation. Patients who had not relapsed at 96 weeks are censored on the time to next bipolar episode outcome. SCID-LIFE interviews to collect time to next relapse are collected every 16 weeks alternating between telephone and face to face interview up to 96 weeks.

Face to face assessments are performed at baseline, 32, 64 and 96 weeks after randomisation. Telephone assessment is used for interviews at 16, 48 and 80 week assessments. At 16, 48 and 80 weeks, self-rated questionnaires are e-mailed or posted using reply paid envelopes as the participant requests. Table 1 shows the schedule of assessment. The assessments by telephone have shown to be valid compared to face to face assessment [49,50]. Some of our measures have not been tested psychometrically by telephone compared to face to face assessment so they are delivered face to face. Face to face assessment is performed if telephone assessment is not feasible. We use case notes and interview key workers to determine time to recurrence if patient consents but drops out from direct follow-up [21,51]; a recurrence is defined as a clinically important change in mental state towards either mania or depression resulting in a substantial change in function and/or necessitating change in treatment or management such as change in medication, urgent care or admission to hospital.

**Table 1:** Measures in the study

Every 16 weeks (up to 96 weeks)	Every 32 weeks (up to 96 weeks)
SCID - LIFE at interview	SAS at interview
HDRS at interview	EuroQol at interview
MAS at interview	Economic Interview (CSRI)
SOFAS at interview	SRMetric - trait at interview
MedAd at interview	SRMetric - diary by post
	HADS version 1 by post
	KAB by post
	EWS checklist by post
	Coping strategies checklist by post
	BIPQ by post
	HIQ by post
	Hayward Stigma Questionnaire by post
	SF-12 by post

### Sample size

When patients receive treatment in groups, interactions between patients may lead to correlation of outcomes of patients in the same group [52] sometimes referred to as cluster. As with cluster randomised trials, sample size calculation needs to consider the possibility of intra-cluster correlation [53]. As there are no previous trials of group interventions for bipolar disorder that have considered clustering, we considered empirical evidence regarding the magnitude of clustering from cluster randomised trials. A previous trial [54] found a negligible clustering effect (0.0001) but this was from a small sample, and so would be imprecisely estimated. We have therefore assumed a small but not zero clustering effect equal to 0.05. Based on the outcomes from the two previous Barcelona protocol psychoeducation randomised controlled trials [7,8] a differential treatment effect of 0.22 was used (60% recurrence in the control group, 38% in the psychoeducation group). Power for 80% probability of detecting a difference at 0.05 level, 2-tailed testing requires 82 patients per arm. Assuming a mean group size of 18 and an intra-cluster correlation coefficient of 0.05, a design effect of 1.85 gives a sample size of 152 in each arm. We have assumed 15% loss to follow up [54] giving a total sample size of 179 per arm (358 in total). This is achieved by running 10 groups of 18 subjects in each arm (10 in the North West and 10 in the East Midlands) over 3 years. Initially 17-18 patents are recruited per group but with attrition there will be 13-14 patients per group, the ideal size for group psychoeducation based on 10 years experience [55].

### Economic evaluation

The relative costs and outcomes of the bipolar group psychoeducation are compared to those of the bipolar group support intervention. The perspective of the evaluation is that of health and social care agencies and patients, which are the key components of a societal perspective. The primary economic outcome measure is the incremental cost per quality adjusted life year (QALY) gained. Resource use data are collected using and economic patient questionnaire (EPQ) which includes questions from the Client Service Receipt Inventory [56] interview and service use questionnaires used in previous mental health trials. Quality adjusted life years will be estimated from the Euroqol (EQ-5D) [34] and published utility tariffs. The EQ-5D has been used successfully in previous trials of psychosis [57,58] and bipolar disorder [35]. The EPQ and Euroqol are completed at baseline, 32, 64 and 96 weeks follow-up. National unit costs are combined with service use data to estimate the direct costs of the interventions.

## Interventions

### Group Psychoeducation

The Bipolar Group Psychoeducation programme is run by three facilitators, two health professionals (usually one experienced and one in training), specially trained for the purpose, and one of a small group of mental health service users known as expert patients [59] trained for the purpose. The bipolar group psychoeducation programme in Barcelona used three therapists over the last 10 years given the large size of the groups [55]. The use of an expert patient in the role of therapist working with a health professional was not tried in Barcelona but was successfully piloted in Newcastle (Dr S Watson, personal correspondence) before the start of the current trial. Feedback suggests the expert patient ensures the service user perspective is integral to the program and provides additional credibility to the programme in the eyes of the participants. The expert patient also serves as a role model for the participants in tasks such as life charting.

In accordance with the Barcelona protocol, the group psychoeducation programme has 21 sessions (see Table 2 for curriculum content of the programme). However, as recommended [55], the content has been brought up to date to reflect recent research evidence [3,5]. The content was adapted to expectations of English service users as stated by a panel of service users working with the trial. For example, the first session includes more content about the nature of bipolar disorder, a greater emphasis on the role of family and the carer, changes to the wording of the manual and handouts, and the English context of service provision. Embedded in this is the acquisition of specific skills by each individual including, life charting, recognition of early warning signs, problem solving and other forms of coping, sleep hygiene and care planning, as well as general skills of actively participating and working collaboratively in the groups.

**Table 2:** Sessions of group psychoeducation treatment

Session number	Topic
1	Introduction to the group and defining bipolar disorder?
2	What causes and triggers bipolar disorder
3	Symptoms 1: mania and hypomania
4	Symptoms 2: depression and mixed episodes
5	Evolution of bipolar disorder and the future
6.	Treatment 1: mood stabilisers
7.	Treatment 2: antimanic drugs
8.	Treatment 3: antidepressants
9.	Pregnancy, genetic counselling and effects on families
10.	Prescribed drugs and alternative therapies
11.	Risks associated with treatment withdrawal
12.	Alcohol, smoking, diet and street drugs
13.	Early detection of mania and hypomania 1
14.	Early detection of mania and hypomania 2
15	Early detection of depression and mixed episodes 1
16	Early detection of depression and mixed episodes 2
17	What to do when a new phase is detected
18	Regularity of habits
19	Stress control techniques
20	Problem solving strategies
21	Finalisation of Stay Well Plan and Closure

The group sessions comprise a closed group ideally starting with 17 or 18 participants. In practice 25% of patients may not attend so the average group size is likely to be 12-14 participants. A manual has been produced with a handout given for each session covering the content of that session. The groups are run in a collaborative workshop with a brief didactic introduction of the topic for the session and the rest of the work taking the form of active interaction using the collective experience of the participants.

Any participant who misses a session will be provided printed materials for the session and an opportunity to discuss the materials before the next session. However, absence of five consecutive sessions is considered a drop-out from treatment and analysed as such when a "per protocol" analysis is performed of participants who adhered to the treatment protocol. Participants who miss occasional sessions are offered a complete set of handouts for these sessions. The work in the psychoeducation group builds on earlier sessions so the involvement of participants who had not attended earlier sessions might be disruptive to the other participants in the group without the opportunity to catch up. The same rules apply to the bipolar group support arm so that there is internal consistency within the RCT to maintain internal validity.

An aim of the bipolar group psychoeducation arm is for each participant to develop their own individualised relapse prevention programme (a "stay well plan"). This includes a list of people that it might be shared with including family members and clinicians, but the final decision on whether to share the care plan rests with the participant. The expert patient and health professionals taking the group are supervised by RM in the East Midlands and FL & SJ in the North West. In addition, the expert patients taking part in the groups can take part in a peer run supervisory group in the North West run by DM. The content and conduct of each session are recorded on written forms firstly by the therapists and then also by the participants (with the therapists not present) using the treatment fidelity checklist. The group sessions have not been audiotaped because if one or more participants did not agree then a session could not be taped.

### Bipolar Group Support treatment arm

The purpose of the Bipolar Group Support intervention is to provide an active control for the bipolar psychoeducation group, which reflects the practice of expert patient and some types of support groups for bipolar disorder in England. The support groups aim to enable the group participants to devise ways of remaining well, through discussion of collective experience, mutual information sharing and support. Although the groups are unstructured, they are peer led and collectively decide upon an agenda for discussion. Thus these groups do not merely provide a meeting place where people with bipolar disorder can meet other people with bipolar disorder but also have a shared sense of purpose to actively seek ways of learning from the group, to remain well in the future. Therefore the Bipolar Group Support not only provides a control for the processes of delivering a group intervention but also in the overall aim of the intervention for each participant, namely to stay well over time. As a result the groups only differ in terms of the content and style of delivery. The two health professionals and one expert patient meet with the groups of up to 18 participants but are there to facilitate discussion, encourage participation, prevent unhelpful group behaviour such as bullying or scapegoating, to prevent factual misinformation, and if directly asked to clear up factual uncertainty. A manual on the conduct of the bipolar support group is produced for the therapists and given as a handout in the first session. The supervision arrangements and recording of the conduct and content of the sessions are the same as for the group psychoeducation sessions.

Both groups of patients will receive the trial group therapies in addition to their usual treatment. The latter is unconstrained and recorded from case notes and at the economic interview.

### Randomisation and treatment allocation

Randomisation is conducted by a clinical trials unit at the University of Nottingham, who will be given the participant information by the programme manager or programme administrator. Randomisation is only undertaken once a group of 20 participants in a wave (figure 1) have been identified, since this is the minimum number that would constitute a viable group size. Maximum group allocation is 36. Allocation of participants to treatment is based on stochastic minimisation using number of previous episodes banded as (<7, 8-19, 20+) and clinical site. The clinical trials unit conveys the allocation to the trial manager who contacts the participants themselves and the therapists conducting the group. The research assistants are blind to group allocation. All information regarding group treatment and cohesion is collected by the therapists so that RAs remain blind. Any subsequent unblindings are recorded by the trial office. Where possible once an RA has been unblinded an alternative RA at that site collects the remainder of that participants follow-up data.

### Qualitative studies

Barriers (attitudinal, experiential and practical) to the effectiveness of group psychoeducation are assessed using audiotaped and transcribed semi-structured qualitative interviews with maximum variance samples of group leaders (health professionals and expert patients) and patients. The transcripts are thematically analysed with reference to a multidisciplinary group. The main focus of the qualitative work relates to the: 1) feasibility of delivering group psychoeducation for relapse prevention in bipolar disorder; 2) key differences in the experience and future relapse prevention care planning of participants in the bipolar group psychoeducation and bipolar group support treatment arms; 3) issues arising from involving expert patients as co-therapists with health professionals (health professionals and expert patients are keeping reflective diaries of their experiences of running the groups); and 4) application of their "stay well plans" after the groups have finished, including their acceptance and help with delivery by health professionals.

### Analysis

Intention to treat using Kaplan-Meier recurrence-free curves with significance tests will be based on the Cox proportional hazards regression model. The level of dependence or heterogeneity among patterns of recurrence

within the same team was estimated by the intracluster correlation coefficient (ICC) using estimate of frailty from the shared frailty model [60]. Trial centre, and number of previous episodes of bipolar disorder, will be included as covariates. Analysis of quantitative secondary outcome measures will use linear mixed models including baseline covariates and random effects for therapy group [52]. The treatment effect may differ according to illness duration/number of episodes with a lesser treatment effect in patients with longer illness duration/more episodes based on findings of the MRC CBT trial [21]. This will be tested in a secondary analysis by adding a severity-treatment interaction into the above models.

The economic analysis will be adjusted for baseline covariates shown to be important predictors of future costs and outcomes (e.g. costs and service use prior to entry, health status and utility, clinical severity and duration of illness, socio-economic status). Bootstrap simulations will be used to estimate cost effectiveness acceptability, net benefit statistics and the likelihood that group psycho-education is cost effective compared to group support. Probabilistic simulation models will be used to explore the generalisability of the results to the UK.

### Ethics and research governance approval

The trial has received national multi centre research ethics approval (09/H0408/33) and research governance approval and permission at each participating health service delivery organisation. Each participant gives written informed consent to the trial and separately also gives written informed consent for the qualitative interviews. The trial is overseen by an independent Trial Steering Committee and Data Monitoring and Ethics committee organised by the research investigators for the purpose. The members of the committee are drawn externally from outside the institutions that the research team currently work to ensure its independence of the research team.

## Results

The study is now recruiting participants across the East Midlands and North West areas of England in the form of a rolling road show visiting centres sequentially.

## Discussion

Although there is evidence from two RCTs of the clinical efficacy of group psychoeducation for bipolar disorder compared to group support in a specialist bipolar disorder service in Spain [7,8] and clinical effectiveness versus treatment as usual in an Australian RCT [12], the evidence base is still relatively thin. In particular, evidence is still required about whether the content and style of group psychoeducation has any specific effect on time to relapse and other clinical and economic outcomes. From a United Kingdom service commissioning perspective, there is a need to establish that group psychoeducation is more clinically and cost effective than the support groups that are quite commonly found in the United Kingdom. These support groups are cheap to fund as they are largely run by charities using expert patients rather than trained professional therapists. Thus this study is designed primarily to establish whether it is the content and style of the intervention delivered in group psychoeducation that is more effective and cost effective than an active control intervention of the same length and duration of treatment, taken by therapists of the same professional background and with the same specific aim of trying to develop a plan of care to stay well over time. However, the study has been adapted to be a pragmatic randomised controlled trial of these interventions to reflect a model that might be used in everyday clinical practice in England. Thus unlike the Barcelona trials, there is an expert patient as a facilitator. In England it is increasingly common to use such patients in this role in England. There may also be advantages in terms of relating the therapists' interventions more closely to patient experience and also costs to health services [15,59]. Furthermore we have used a range of different health professionals to reflect clinical practice in England rather than only psychiatrists and clinical psychologists as in the Barcelona trials.

Seven features of trial design and conduct may distinguish a pragmatic RCT from an efficacy or more theoretically driven RCT [61]. On such a continuum, our current RCT has more features of a pragmatic RCT than an efficacy RCT but some compromises have been made so that it can achieve its primary objective, which is arguably primarily theoretical. Thus the study question has a theoretical component to it and as such the control intervention in terms of length and duration of treatment and professional background of the therapists does not reflect the reality of most expert patient groups providing support for bipolar disorder in United Kingdom. They are run by expert patients rather than professional therapists, are either closed and run for fewer sessions or open ended and open to new membership, and may have some content similar to that found in the group psychoeducation intervention.

On the other hand a pragmatic RCT design approach has been taken in terms of other features of the study design. Broad inclusion/exclusion criteria of the group participants have been applied with exclusions applying only to lack of clarity around diagnosis or an inability to participate in the intervention as would be required in routine clinical practice, where access to interventions and generalisability of findings are a primary concern. Hence the setting is not a specialist bipolar disorder service with highly trained therapists [7,8], but routine health services using therapists with limited experience of the specific interventions, but with clinical or personal experience of bipolar disorder and often previous experience of delivering psychological interventions. The group psychoeducation intervention has been adapted from the content of the published manual [55], both to keep it up to date with a fast changing field of evidence based practice but also on the advice of a panel of service users and health professionals, some changes to the content of some of the sessions and the materials or scenarios used to fit well with an English clinical setting. For instance, the first session might not engage English patients if it did not directly tackle the nature of bipolar disorder and was restricted to introductions and rules about running the group. There is also a greater emphasis on the family, less need to tackle ideas from established religion, a need to consider English service structures e.g. crisis resolution and home treatment teams and community mental health teams [20], and how they could be utilised with a more extended early warning signs intervention.

The main strengths of the RCT are the size of the sample and its multi-site design allowing consistency of intervention effects and their generalisability to be explored. Unlike a psychological treatment study compared against treatment as usual or a briefer intervention, it may be possible to achieve independent and blinded rating of outcome because both interventions are similar in form and duration but differing in content. Outcomes are assessed using well-validated measures that we have applied successfully before in a series of RCTs on psychological interventions in bipolar disorder. Furthermore although the trial has a clear theoretical aim, it is carried out using methods that are relatively close to usual clinical practice settings, again allowing the results to be readily generalisable to routine clinical practice. The combination of qualitative and quantitative data collection around mechanisms of action, the nature of the intervention and its effects, and barriers to its delivery allows sufficient detail both to replicate these complex interventions and optimise their delivery for both further research and clinical practice [62].

The main weakness of the current RCT is the lack of a treatment as usual group. Whilst the trial will report on the relative clinical and cost effectiveness of the two interventions it cannot definitively show that either is more clinical or cost effective than treatment as usual. The process measures will be able to track whether key processes are changing in the groups, particularly in the group psychoeducation intervention. Thus, in both groups there should be evidence of group cohesion if these interventions are likely to be effective but in addition the specific content of the group psychoeducation intervention should show improvements in knowledge about bipolar disorder, early warning signs and adaptive strategies, and regularity of social rhythm coping. If such changes are not happening over time in the participants who have adhered to the group psychoeducation, then it is less likely that group psychoeducation had been effective in this RCT. While group psychoeducation has demonstrated improvements in medication adherence [63], previous psychological treatment studies performed by our group [21,51] have not demonstrated such benefits because of ceiling effects with high levels of medication adherence in both intervention and treatment as usual groups.

Other potential weaknesses include uncertainty concerning the design effect of the study due to clustering and potential difficulties in recruitment because the onset of the study is delayed until sufficient size groups are recruited. Both these factors may adversely affect the sample size needed to show a true difference in effectiveness between the two treatment groups. The relative inexperience of the therapists may also dilute the effectiveness of both treatments although they may reflect a more accurate picture of their effectiveness in routine clinical practice settings.

In terms of research implications, the trial will provide a rigorous test of whether the content and style of group psychoeducation similar to that provided in the trials from Barcelona is effective. If so the trial will produce more evidence concerning its mode of action. From a clinical implementation perspective, the cost effectiveness of the interventions and barriers and drivers to the delivery of the intervention will enable service providers to decide whether the intervention is worth providing and also how best to deliver it.

## Competing interests

The authors declare that they have no competing interests.

## Authors' contributions

RM is a grant holder, joint principal investigator of the study, is responsible for the conduct of the study in the East Midlands area of England and measurement of mental state outcome in the study and wrote the first draft of the paper. FL is a grant holder, joint principal investigator of the study, is responsible for the conduct of the study in the North West area of England. SJ is the Chief Investigator for the PARADES Programme of Research and responsible for the measurement of processes in the study. LR is the study co-ordinator and for the PARADES Programme. SP leads the qualitative parts of the study and is a grant holder. CR is the trial statistician and is a grant holder. LD leads the economic analysis and is a grant holder. DM leads the analysis from a service user perspective and is a grant holder. All authors contributed to the design of the study, revised the manuscript and gave final approval to the manuscript.

## Pre-publication history

The pre-publication history for this paper can be accessed here:

http://www.biomedcentral.com/1471-244X/11/114/prepub
